# Face-to-Face and Internet-based Cognitive Behavioral Therapy for Patients with Heart Failure: an Umbrella Review of Systematic Reviews and Meta-analyses

**DOI:** 10.1007/s11897-026-00760-1

**Published:** 2026-05-09

**Authors:** Huinan Liu, Zhixiang Peng, Wai Kai Hou

**Affiliations:** 1 Department of Special Education and Counselling, The Education University of Hong Kong, Tai Po, Hong Kong SAR China; 2 Centre for Psychosocial Health, The Education University of Hong Kong, Tai Po, Hong Kong SAR China; 3 Department of Psychology, The Education University of Hong Kong, 10 Lo Ping Road, Hong Kong SAR, Tai Po, NT China

**Keywords:** Heart failure (HF), Cognitive behavioral therapy (CBT), Umbrella review, Meta-analysis

## Abstract

**Purpose of review:**

The present study aims to provide an up-to-date umbrella review of existing meta-analyses of randomized control trials (RCT) of cognitive behavioral therapy (CBT) among patients with heart failure (HF).

**Recent findings:**

The umbrella review covered five meta-analyses published in the past five years with 18 primary studies (conducted between 2010 and 2022) of 1,687 non-duplicated patients aged 51.63 to 77.40. Primary studies originated from the USA, Sweden, Netherlands, China, Iran, and Philippines. Common benefits of CBT included depressive symptoms (89%), quality of life (56%), anxiety symptoms (39%), self-care (33%), 6-minute walk distance (33%), and health status (17%). Evidence varied depending on the timepoint of follow-up assessment and the specific measurement used to evaluate the outcome. No meta-analyses pooled the effect sizes for iCBT and analyzed the specific therapeutic elements in CBT. No meta-regression was performed.

**Summary:**

Individual participant data meta-analyses with meta-regression should be performed to identify common moderators and factors that can influence treatment adherence. The current research would also benefit from additional RCT evidence focusing specifically on iCBT for patients with HF.

**Supplementary Information:**

The online version contains supplementary material available at 10.1007/s11897-026-00760-1.

## Introduction

Heart failure (HF) is a chronic and debilitating condition of diminished cardiac output or increased pressure within the heart resulting from structural or functional heart impairments [1]. HF represents a major global public health challenge with an estimated 38 million patients worldwide especially among individuals aged 65 or above [2]. A recent global review of HF prevalence revealed that one-year mortality rates ranged from 4% to 45%, with an overall average of 33% and a slightly lower average of 24% in studies involving all adult age groups [3]. HF is typically categorized into three primary types, namely preserved (≥ 50%), mid-range (40%–49%), or reduced (≤ 40%) ejection fraction. Driven by the aging of global population, the prevalence continues to surge and there is also a notable increase in younger adults with a shift towards HF with preserved ejection fraction [2, 4]. In addition, despite substantial treatment advancement, the prognosis for HF is worse than other chronic medical conditions such as most cancers [5].

Depression and anxiety are prevalent comorbid psychological conditions among patients with HF. Patients with comorbid psychiatric symptoms showed increased rate of healthcare utilization, hospitalization, and mortality [1]. Depression has been independently associated with adverse cardiac outcomes over and beyond other cardiovascular risk factors [5]. Depression was associated with reduced self-care practices of HF patients, such as maintaining an active lifestyle and adhering to a low-sodium diet, which in turn related to poorer physical functioning [6]. A scoping review comparing psychiatric and psychological interventions for depression in patients with heart diseases revealed that, unlike those with coronary artery disease, HF patients show limited evidence of benefiting from selective serotonin reuptake inhibitors (SSRIs). Instead, these patients appear to be more responsive to psychotherapy [5].

As a widely used gold-standard psychotherapy, Cognitive behavioral therapy (CBT) relative to pharmacological intervention has demonstrated distinct benefits on cardiac outcomes and quality of life through enhancing self-care skills such as adherence to prescribed medications and commitment to recommended dietary and exercise regimens [1, 7]. Meta-analytic evidence has also showed the positive impact of CBT on ameliorating physical and psychological distress across chronic medical conditions [8]. Traditional face-to-face CBT with trained therapist is constrained by challenges such as restricted access, lack of scalability and feasibility [7, 9]. Internet-based CBT (iCBT), an online evidence-based therapy offers text, video, and audio content comparable to face-to-face CBT in duration and content, are available either with therapist guide or as self-guided modules [10]. iCBT addresses core limitations of face-to-face CBT, including extended waiting periods, accessibility barriers, scalability, high costs, and disparities in healthcare resources [11]. Therapist-guided iCBT has also been shown to be equally effective in treating depression and anxiety compared with in-person therapy [12, 13].

Currently only limited evidence is available to show the physical and mental health benefits of iCBT among cardiac patients. The most recent meta-analysis of 1,177 patients with cardiovascular and cerebrovascular diseases found that iCBT can significantly reduce both anxiety and depressive symptoms, with self-guided iCBT outperforming therapist-guided iCBT, and programs with fewer modules and shorter durations being more effective for anxiety while programs with more modules and longer durations are more effective for depressive symptoms [9]. However, only one randomized controlled trial involving patients with HF was included in the analysis. Despite the heterogeneity among the cardiovascular disease patient population, no subgroup analysis was performed to investigate the benefit of iCBT specifically on HF patients in previous systematic reviews and/or meta-analyses. Another systematic review examined a range of outcomes associated with CBT among patients with HF, including sleep, quality of life, depression, and anxiety [14]. However, the review was limited by the use of outdated references published between 2010 and 2020 and did not differentiate the treatment effects of iCBT from other delivery modalities of CBT. Other existing review studies have either been limited in their scope [5, 15, 16], focusing solely on specific outcomes associated with CBT (e.g., depression), have failed to examine the internet-based delivery of CBT [1, 17–20], targeted at specific therapeutic element of CBT [21, 22], or only included studies of specific geographic region [23].

Although multiple meta-analyses have been conducted in recent years, they differed substantially in inclusion criteria, CBT modalities, outcome measures, and follow-up timepoints. These methodological discrepancies have contributed to the inconsistent findings. Notably across the five meta-analyses published in the past five years (2021-March 2026) which collectively included 18 non-duplicated primary randomized control trials, only two primary studies [24, 25] overlapped across the five reviews. Ten of the 18 primary studies appear in only a single meta-analysis, indicating that each meta-analysis synthesized a distinct body of empirical evidence and that the pooled effect sizes reported were not simply re-analyse of the same dataset. This highlights the need to integrate the available evidence of outcome-specific and timepoint-specific patterns of CBT treatment effects and elucidate how differences in study context and implementation may influence the findings. Furthermore, a systematic synthesis of recent meta-analytic review, particularly those based on randomized controlled trials, provides an efficient way of identifying the critical research gap in the field. Given the reciprocal relationship between psychological and physical symptoms in patients with heart failure, a comprehensive understanding of the effectiveness of CBT is necessary to inform clinical prioritization of intervention.

The present study aims to provide an up-to-date umbrella review of existing meta-analyses of randomized control trials of CBT among patients with HF. We included meta-analyses of RCT studies in the past five years to investigate (i) the psychological (e.g., depressive symptoms) and physical (e.g., cardiac benefit, hospitalization, mortality rates) benefits of CBT among patients with HF; (ii) the therapeutic elements of the CBT; and (iii) whether iCBT was more effective than face-to-face CBT. The current study also investigated the common moderators for the effectiveness of CBT (e.g., session number, type of HF) and factors that promote treatment adherence among patients with HF.

## Methods

### Search Strategy and Selection Criteria

The keywords search was conducted to explore the use of CBT for psychological and physical outcomes among patients with heart failure. We then used, a population, intervention, comparison and outcome (PICO) framework to refine research question and guide search strategy (Table 1). Based on these components, the primary research question guiding the literature search was: Among patients with heart failure, whether or not CBT improves psychological and physical outcomes compared with the control conditions. As the initial search indicated substantial existing evidence base, the search strategy restricted to studies of the highest level of evidence and focused on systematic reviews and meta-analyses of randomized controlled trials. We adhered to the PRISMA guidelines and separately searched four databases, namely PsycINFO, Medline, PubMed, and Web of Science to identify review articles published in English within the last five years, from 2021 up to March 31 st, 2026. The search utilized a combination of keywords spanning three distinct categories, namely *heart failure* [Heart Failure] *psychotherapy* [Psychotherapy OR Psychotherap* OR CBT OR Cognitive behavioral therapy OR Behavioral therap* OR Behavioral activation* OR Behavioral strateg* OR Behavioral intervention* OR Behavi* OR Exposure therap* OR Cognitive therap* OR Cognitive intervention* OR Cognitive reappraisal OR Cognitive restructur* OR Cognitive strateg* OR Cogni* OR Psychoeducation], and *randomized control trial* [RCT OR randomized controlled* OR randomized clinical* OR randomized placebo-controlled* OR randomized trial*]. To be more inclusive and avoid the case where meta-analysis were not reported in the title, we did not include the keyword ‘meta-analysis’ in our search strategy and instead manually screen the full texts. The PRISMA checklist was included in the Appendix A.

**Table 1:** PICO Framework

*P*-Patient or population	Adults diagnosed with heart failure
I-Intervention	CBT or CBT-based interventions (internet-based or face-to-face)
C-Comparison	Usual care, waitlist controls, active controls
O-Outcome	Psychological: depressive symptoms, anxiety symptoms, quality of life, etc. Physical: cardiac benefits, health status, hospitalization, mortality rates, etc.

Eligible studies included systematic reviews with meta-analyses of randomized controlled trials that provided quantitative synthesis of the pooled estimates on the effectiveness of CBT among patients with HF. Exclusion criteria included (i) systematic reviews without meta-analyses giving our aim to conduct quantitative comparisons and evaluate study quality; (ii) meta-analysis published beyond five years. We did not limit scope of outcome types to provide a thorough evaluation of the potential treatment targets of CBT in this population.

**Fig. 1:**
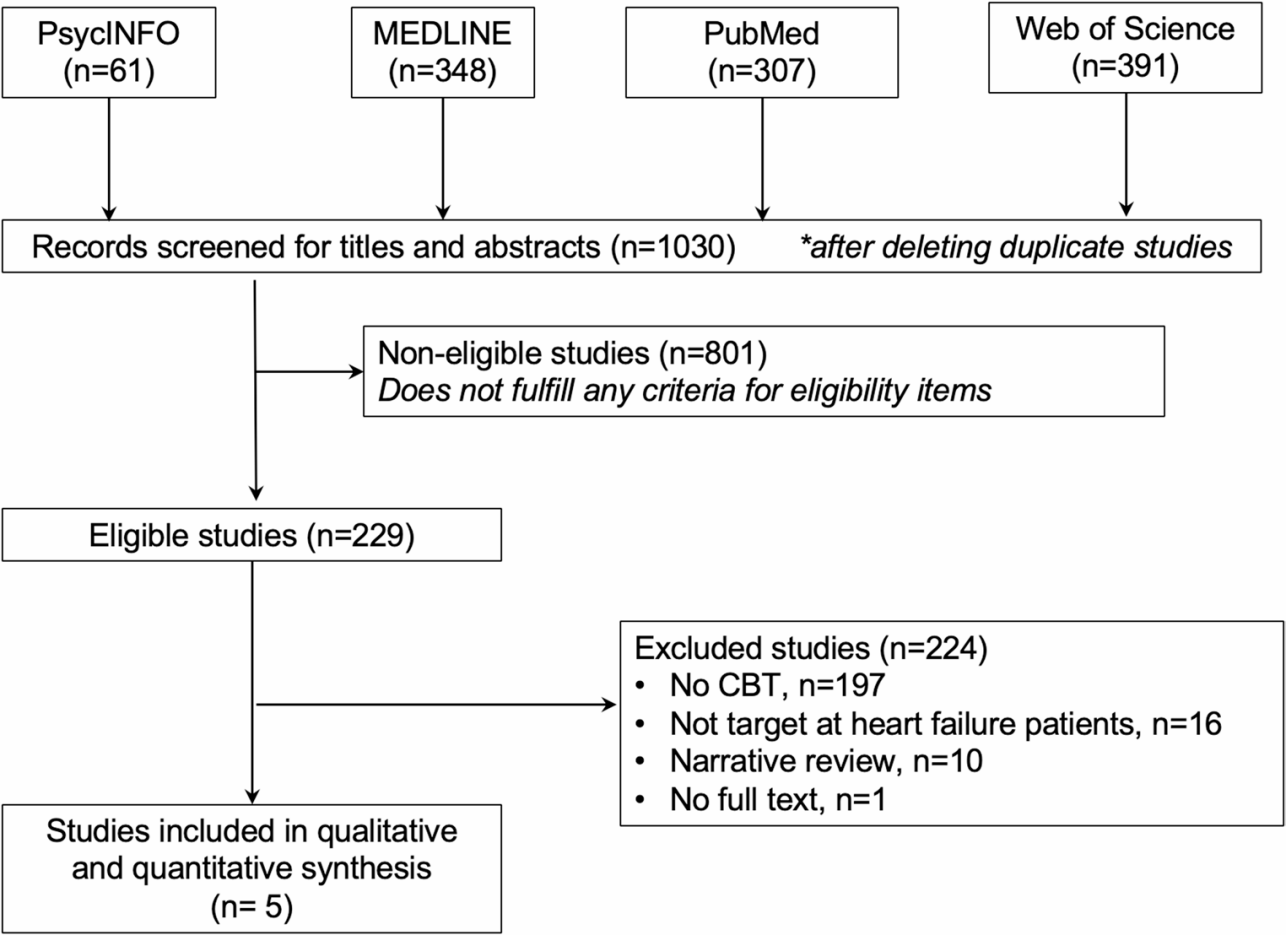
Study flowchart

The data extraction commenced on March 31 st, 2026. In the initial stage, two independent reviewers (HL and ZP) screened the titles and abstracts to assess eligibility, with any discrepancies discussed among all authors and resolved prior to progressing to the next stage. In the second stage, two independent reviewers/authors (HL and WKH) reviewed the full-text articles and extracted the relevant data for the qualitative synthesis. The eligibility of each included study was double-checked by a second reviewer (WKH), and any disagreements were resolved through discussion and reiteration of the data extraction. The umbrella review was not registered and study flow was visualized in Fig. 1. This article does not contain any studies with human or animal subjects performed by any of the authors.

### Data Analysis

A standardized data extraction form was utilized by three independent reviewers/authors (HL, ZP, and WKH) to systematically extract the relevant information from the included studies.

Study characteristics included country and year of publication of primary studies, sample sizes included in the meta-analysis (k), pooled sample size (n1 for intervention group, n2 for control group), age and female distribution of the samples, and pooled effectiveness of CBT with 95% and corresponding heterogeneity statistic (I^2^) from random-effects meta-analysis. The I² statistic was used to quantify the proportion of variability; 50% or below indicated low heterogeneity. We used a descriptive approach to synthesize findings from the included meta-analyses. Estimates on the pooled effectiveness (along with the 95% CIs and I^2^ statistic) and results from meta-regression analyses in the respective meta-analyses were reported.

### Quality Assessment

Risk of Bias in Systematic Reviews (ROBIS) instrument was used to evaluate the methodological quality of the included meta-analyses. The ROBIS instrument comprises four key domains: study eligibility criteria, identification and selection of studies, data collection and study appraisal, and synthesis and findings. Overall scores on risk of bias were calculated and classified as low, moderate, or high risk of bias [26, 27].

## Results

The initial search yielded 1,030 records from four databases (PsycINFO, MEDLINE, PubMed, and Web of Science) after removing duplicate entries. 801 studies were excluded in the first stage screening that did not fulfill the eligibility criteria. The remaining 229 eligible studies were assessed for eligibility, with 224 studies further excluded for the following reasons: 197 studies were not relevant to CBT, 16 studies did not target HF patients, 10 studies were narrative review without meta-analysis, and 1 study did not have full text. Finally, five systematic reviews and meta-analyses were included for qualitative synthesis. Full selection process was visualized in Fig. 1. Study characteristics were summarized in Table 2.

**Table 2:** Study characteristics

Meta-analysis	k	Outcome	Countries/Regions (% of HICs)	Database	*n*	Date range	Designs	Follow-up Range	ES	Age	Female percentage	ES on iCBT	Tools for RoB	Meta-regression	Percentage of NYHA class III or IV	Percentage of ischemic HF	Publication bias
Chernoff et al. (2022) [28]	10	Depression, Anxiety, QoL, Hospitalization, Mortality	90% (6 USA, 1 Sweden, 1 Taiwan,1 Iran 1 Netherlands)	PubMed, Ovid MEDLINE, PsycINFO, and Cochrane database	1370	1970–2021	RCTs	4–52 weeks	DSMC	51.6–77.4	39.0%−39.2%	No	Cochrane Risk of Bias tool	Yes	35.4%	NR	Indication of publication bias
Balata et al. (2023) [29]	7	Depression, Anxiety, Self-care, Health status	57.14% (4 USA, 2 Iran, and 1 Philippines)	PubMed, Scopus, Web of Science, and Cochrane Library	611	2010–2022	RCTs	4–9 months	SMD	51.63–68.0.63.0	31%−75%	No	Cochrane Risk of Bias tool	No	26.19%	NR	Not assessed
Nso et al. (2023) [30]	9	Depression, Anxiety, QoL, Self-care, 6 min walking distance	77.78% (6 USA, 1 Sweden, 1 Iran, and 1 China)	PubMed, Scopus, Web of Science	1070	2014–2020	RCTs	3–6 months	SMD	55.5–74.6	18.5% − 58.1%	No	Cochrane Risk of Bias assessment tool	No	NR	NR	NR
Mhanna et al. (2023) [31]	6	Depression, Death Anxiety, QoL, Self-care, 6 min walking distance	83.33% (4 USA, 1 Iran, and 1 Sweden)	PubMed/MEDLINE, EMBASE, Cochrane	489	2010–2022	5 RCTs & 1 Quasi-experimental study	1 week to 12 months	SMD	58.3–59.0	45%−47%	No	Jadad scale	No	45%	42%	No publication bias
Soleimani et al. (2024) [32]	5	Depression	80% (4 USA, 1 Iran)	PubMed, Scopus Web of Science	492	2000–2015	RCTs	8 weeks − 24 months	SMD	52.3–66.0	NR	No	Cochrane Risk of Bias tool	No	NR	NR	No publication bias

k= number of studies; n=number of patients; *DSMC* difference in standardized mean change, *ES* effect size, *RoB* risk of bias, *QoL* Quality of life, *RCT* Randomized control trial, *SMD* Standardized mean difference, *NYHA* New York Heart Association

The present umbrella review covered five meta-analyses involving 18 primary studies of 1,687 non-duplicated patients published between 2010 and 2022 (Primary empirical studies included in the five meta-analyses were listed in Appendix B). The study samples aged between 51.63 and 77.4 years, with female percentage ranging from 18.5% to 75%. Percentage of NYHA class III or IV ranged from 18.5% to 75%, and percentage of ischemic HF ranged from 13.46% to 42%. Primary studies originated from the USA, Sweden, Netherlands, China, Iran, and Philippines. The proportion of study samples from high-income countries ranged from 57.14% to 90%. Of the meta-analyses on psychological outcomes, 89% (16 out of 18) of the underlying samples were on depressive symptoms 56% on quality of life, and 39% on anxiety symptoms. For physical/behavioral outcomes, 33% (6 out of 18) of the primary samples were on self-care, 33% on 6-minute walk distance, and 17% on health status.

Almost all meta-analyses adopted standardized mean differences as the effect sizes (see full results in Table 3). Heterogeneity (I^2^) ranged from 0% to 94% for psychological domain and 0% to 95% for physical domain. The overall effect estimates favored CBT over control group for depressive symptoms at post-intervention and 3-month follow up [15, 19, 33, 34]. At post-intervention, three meta-analyses found small to moderate effect in favor of CBT over control conditions (SMD = − 0.58 to −0.37) though substantial heterogeneity was observed (I² = 37%−76.3%) [15, 33, 34]. Small but significant reductions of depressive symptoms were observed at three months (SMD, − 0.18 [− 0.33 to − 0.02]), though the effect at six months was no longer significant with substantial heterogeneity (I² = 94%). However, no significant difference was found at less than 12 weeks follow-up (SMD, 4.34 [−10.70 to 2.03]) [17] and treatment effect at 4-to-9-month follow-up were mixed depending on scale used for measuring depressive symptoms [19]. In contrast, the beneficial effects of CBT on depressive symptoms was found to be consistently sustained from one week to 12 months follow-up in one review [15]. Balata et al. (2023) conducted separate analyses of depressive symptoms using two instruments, Beck Depression Inventory-II (BDI-II) and the Hamilton Rating Scale for Depression (HRSD-17). For primary studies using BDI-II, the effect sizes were large but not significant at less than 12 weeks of follow-up. However, the therapeutic effects of CBT strengthened overtime and became. significant at four to six months (SMD = − 4.87, [− 8.06, − 1.69]) and at eight to nine months (SMD = − 5.71, [− 8.95, − 2.46]). In contrast, primary studies using HRSD-17 consistently demonstrated small and non-significant effect sizes across all follow-up timepoints. Improvements on anxiety symptoms were evident at post-treatment (SMD, −0.3 [−0.51 to −0.08]), 4-to-6-month follow up (SMD, −3.54 [−6.64 to −0.45]) and 8-to 9-month follow-up (SMD, −3.9 [−7.05 to −0.74]) [17] but were not significant at less than 3 months follow-up and at 6-month follow-up [17, 19, 34]. Quality of life had significant improved at post-treatment, 3-month, 6-month, and 12-month follow-up, though the heterogeneity in these meta-analyses were all 0% [15, 19, 34]. For physical domain, the pooled effect estimates did not indicate a statistically significant advantage of cognitive-behavioral therapy (CBT) over the control condition for self-care outcomes within the 6-month follow-up period. However, one meta-analysis reported a significant improvement in confidence related to self-care at the 8- to 9-month follow-up (SMD, 9.43 [3.12 to 15.73]). Improvement on 6-minute walk distance were evident in one meta-analysis though it did not specify the timepoint for follow-up (SMD, 5.64 [14.62 to 56.65]) [19]. The short-term effects on health status did not demonstrate statistical significance prior to the 6-month follow-up assessment. However, the improvements in health status were found to be statistically significant at the 8- to 9-month follow-up time (SMD, 9.43 [3.12 to 15.73]) [17]. One meta-analysis narratively examined hospitalization and mortality outcomes without pooling the effect sizes and reported lower hospitalization, mortality rate, and higher cardiac event-free survival rate in CBT group compared to patients with usual care [34].

**Table 3:** Effectiveness of CBT, by domains

	Meta-analysis	Timepoint of follow-up	k	CBT vs. Control (n1 vs. n2)	Statistics	Effect size (95%)	I²
Psychological domain							
Depression							
Depression	Chernoff et al. (2022) [28]	Post-intervention	10	504 vs. 437	DSMC	**−0.37 [− 0.70**,** − 0.05]**	76.3%
Depression	Soleimani et al. (2024) [32]	Post-intervention	5	250 vs. 250	SMD	**−0.58 [− 1.00**,** − 0.12]**	NR
Depression	Mhanna et al. (2023) [31]	Post-intervention	6	**244 vs. 245**	SMD	**−0.45 [−0.69**,** −0.21]**	37%
Depression	Nso et al. (2023) [30]	3 months	6	323 vs. 323	SMD	**−0.18 [− 0.33 to − 0.02]**	0%
Depression	Nso et al. (2023) [30]	6 months	3	194 vs. 194	SMD	−0.78 [− 1.7 to 0.13]	94%
Depression -BID-II	Balata et al. (2023) [29]	< 12 weeks follow-up	5	241 vs. 243	SMD	−4.34 [−10.70 to 2.03]	95%
Depression -HRSD-17	Balata et al. (2023) [29]	< 12 weeks follow-up	2	148 vs. 149	SMD	−0.55 [−4.72 to 3.62]	0%
Depression -BID-II	Balata et al. (2023) [29]	4 to 6 months follow-up	2	148 vs. 149	SMD	**−4.87 [−8.06 to −1.69]**	0%
Depression -HRSD-17	Balata et al. (2023) [29]	4 to 6 months follow-up	2	148 vs. 149	SMD	−2.3 [−6.52 to 1.91]	45%
Depression -HRSD-17	Balata et al. (2023) [29]	8 to 9 months follow-up	2	148 vs. 149	SMD	−1.48 [−5.70 to 2.75]	47%
Depression -BID-II	Balata et al. (2023) [29]	8 to 9 months follow-up	2	148 vs. 149	SMD	**−5.71 [−8.95 to −2.46]**	0%
Depression	Mhanna et al. (2023) [31]	1 week to 12 months follow-up	5	**433 in total**	SMD	**−0.68 [−0.87**,** −0.49]**	0%
Anxiety							
Anxiety	Chernoff et al. (2022) [28]	Post-intervention	6	403 vs. 337	DSMC	**−0.30 [− 0.51**,** − 0.08]**	28.2%
Anxiety	Nso et al. (2023) [30]	3 months	4	292 vs. 293	SMD	−0.04 [− 0.26 to 0.17]	43%
Anxiety	Nso et al. (2023) [30]	6 months	2	154 vs. 154	SMD	0.07 [− 0.36 to 0.50]	73%
Anxiety-BID-I	Balata et al. (2023) [29]	< 12 weeks follow-up	2	148 vs. 149	SMD	−1.78 [−4.87 to 1.31]	0%
Anxiety-BID-I	Balata et al. (2023) [29]	4 to 6 months follow-up	2	148 vs. 149	SMD	**−3.54 [−6.64 to −0.45]**	0%
Anxiety-BID-I	Balata et al. (2023) [29]	8 to 9 months follow-up	2	148 vs. 149	SMD	**−3.9 [−7.05 to −0.74]**	0%
Quality of life							
Quality of life	Chernoff et al. (2022) [28]	Post-intervention	6	367 vs. 312	DSMC	**0.20 [0.04**,** 0.36]**	0.0%
*Quality of life*	Nso et al. (2023) [30]	3 months	3	243 vs. 244	SMD	**4.92 [1.14 to 8.71]**	0%
Quality of life	Nso et al. (2023) [30]	6 months	2	154 vs. 154	SMD	**7.72 [0.77 to 14.68]**	0%
Quality of life	Mhanna et al. (2023) [31]	1 week to 12 months follow-up	4	367 in total	SMD	**0.45 [−0.65 to −0.24]**	0%
Physical domain							
Self-care	Nso et al. (2023) [30]	3 months	3	127 vs. 125	SMD	8.52 [− 7.52 to 24.57]	95%
Self-Care	Mhanna et al. (2023) [31]	2 to 12 months follow-up	2	148 vs. 149	SMD	0.17 [−0.08 to 0.42]	14%
SCHFI Maintenance	Balata et al. (2023) [29]	< 12 weeks follow-up	2	148 vs. 149	SMD	1.2 [−5.24 to 7.64]	25%
SCHFI Maintenance	Balata et al. (2023) [29]	4 to 6 months follow-up,	2	148 vs. 149	SMD	3.43 [−3.03 to 9.88]	0%
SCHFI Maintenance	Balata et al. (2023) [29]	8 to 9 months follow-up	2	148 vs. 149	SMD	−0.31 [−6.84 to 6.23]	0%
SCHFI Confidence	Balata et al. (2023) [29]	< 12 weeks follow-up	2	148 vs. 149	SMD	4.9 [−1.29 to 11.09]	0%
SCHFI Confidence	Balata et al. (2023) [29]	4 to 6 months follow-up	2	148 vs. 149	SMD	4.72 [−1.37 to 10.81]	0%
SCHFI Confidence	Balata et al. (2023) [29]	8 to 9 months follow-up	2	148 vs. 149	SMD	**9.43 [3.12 to 15.73]**	0%
6-minute walk distance							
6-minute walk distance	Nso et al. (2023) [30]	NR	4	152 vs. 148	SMD	**5.64 [14.62 to 56.65]**	0%
6-minute walk distance	Mhanna et al. (2023) [31]	3 to 12 months follow-up	2	148 vs. 149	SMD	0.45 [−0.39 to 1.28]	77%
Health status							
KCCQ	Balata et al. (2023) [29]	< 12 weeks follow-up	2	148 vs. 149	SMD	4.90 [−1.29 to 11.09]	0%
KCCQ	Balata et al. (2023) [29]	4 to 6 months follow-up	2	148 vs. 149	SMD	4.72 [−1.37 to 10.81]	0%
KCCQ	Balata et al. (2023) [29]	8 to 9 months follow-up	2	148 vs. 149	SMD	**9.43 [3.12 to 15.73]**	0%
Hospitalization	Chernoff et al. (2022) [28]	Varied and not possible to pool the effect size	5	NR	Narrative	5/5 studies favoured CBT	NR
Mortality	Chernoff et al. (2022) [28]	Varied and not possible to pool the effect size	5	NR	Narrative	2/5 studies showed benefit	NR

*BDI-II* Beck Depression Inventory, *BDI-I* Beck Anxiety Inventory, *HRSD-17* Hamilton Rating Scale for Depression; *KCCQ* Kansas City Cardiomyopathy Questionnaire; *NR* Not reported, *QoL* Quality of Life, *SCHFI* Self-Care of Heart Failure Index, *SMD* Standardized mean difference

Cochrane Risk of Bias assessment tool and Jadad scale were used in meta-analyses to evaluate risk of bias for the primary studies. Two meta-analyses did not report publication of bias, two reported evidence of publication bias and one reported no publication bias. Quality assessment of all included reviews was summarized in Appendix C. For ROBIS, all five meta-analyses were rated as low risk of bias. Common limitations were absence of efforts made to minimize error in risk of bias assessment and lack of efforts in addressing between-studies variation. Additionally, no meta-analyses were found that independently pooled the effect sizes for iCBT and specifically analyze the specific therapeutic elements in CBT. The available meta-analyses did not employ meta-regression to explore the potential sources of heterogeneity across the included studies and did not discuss factors that can promote treatment adherence among patients with HF.

## Discussion

The present umbrella review of five meta-analyses published in the past five years provided a comprehensive overview of RCT evidence on the effectiveness of CBT among patients with HF in recent years. The majority of the evidence was from middle-aged and older adults in high-income countries. Common psychological outcomes of CBT included depressive symptoms, anxiety symptoms, and quality of life, while common physical outcome included self-care, 6-minute walk distance, and health status. Evidence varied depending on the timepoint of follow-up assessment and the specific measurement used to evaluate the outcome. Depressive symptoms exhibited pronounced improvements at post-intervention and 3-month follow-up, while evidence for anxiety symptoms improvements was more fluctuating, with stronger findings at the 4-to-6-month and 8-to-9-month follow-up assessments. Quality of life steadily improved at 3-month, 6-month, and 12-month follow-up. For physical/behavioral outcomes, our umbrella review suggested that CBT cannot improve overall self-care capability in patients with HF but can improve confidence in self-care at 8- to 9-month follow-up. CBT was found to have delayed benefits for health status, with more pronounced effects observed at the 8-to-9-month follow-up compared with the short-term impacts prior to the 6-month follow-up period.

Although the five meta-analyses were published within a similar timeframe, among the 18 non-duplicated primary randomized controlled trials identified from inception to March 2026, only two RCTs were commonly included across all five reviews. This limited overlap suggests that differences in the contexts and intervention implementation were the potential sources of variability in the research findings. First meta-analysis incorporating fewer primary studies [15, 35] tended to report larger effect sizes than those involving a greater number of trials [34]. Second the source of heterogeneity also originated from the use of different outcome measurement instruments. In particular, therapeutic effects of CBT measured by BDI-II was found to be larger and more sustained, whereas those measured by HRSD-17 were consistently small and statistically non-significant [17]. Third, meta-analysis with a higher proportion of high-income countries reported more favorable treatment effects compared with those that included a greater representation of low- and middle-income countries, though future individual participant data meta-analyses are encouraged to triangulate the source of heterogeneity. Last but not least, CBT modality particularly in studies with large sample size can influence the pooled effect size by carrying disproportionate weight in the meta-analysis. Notably, the largest primary study involving 317 patients [36] was only included in one review [34]. Rather than employing therapist-delivered CBT, this RCT implemented a nurse-led self-management group programme, which may partly explain the relatively smaller effect sizes on depressive symptoms.

### Reciprocity Between Physical Symptoms and Psychological Symptoms

The delayed benefits on confidence in self-care and overall physical health status could be explained by the improvement in psychological outcome (i.e., anxiety symptoms, depressive symptoms, and quality of life) at early stages. The observed reciprocal relationship between physical and psychological symptoms among HF patients aligns with the findings of a recent meta-analysis on internet-based and mobile-based CBT, which demonstrated that changes in psychiatric symptoms and physical distress prospectively predict each other over time in chronic patients [8]. An empirical study of CBT among CVD patients consistently found that anxiety symptoms were significantly related to CBT prognosis via engagement in health prevention behaviors and reducing major adverse cardiac events [26]. Additionally, another study also found that improvements in depressive symptoms were associated with an autonomous-based approach to self-care, which is a crucial factor for enhancing symptom prognosis in HF patients [27]. Given the potential benefits of CBT on cardiac outcomes for HF patients, accessibility to psychological treatment remains an unmet need in cardiovascular disease (CVD) management. Research has shown that psychological treatment is not routinely provided as a standard component of cardiac care, with only 25% of CVD patients eligible for cardiac rehabilitation programs, which also face a shortage of trained CBT therapists [26, 33].

### Internet-based CBT

To address the knowledge deficit about subgroup analyses to assess the efficacy of internet-based cognitive behavioral therapy (iCBT), we extracted all five empirical studies on iCBT from the reference list for further examination. Four of these studies originated from the same research project in Sweden, which was a two-arm RCT comparing a 9-session, 7-module iCBT program to an active control of an online discussion forum on HF self-care. The fifth study was a research protocol for a commercially available digital CBT program called Daylight in the United States [26]. We summarize the descriptive data for iCBT in Appendix D. Similar to findings from face-to-face CBT, results from the Sweden studies did not reveal any statistically significant group differences in self-care outcomes between iCBT and control group, at both the 3-week and 9-week follow-up. However, the improvement in depressive symptoms was found to be significantly associated with improvements in autonomy-related self-care behaviors. Benefits of iCBT extend beyond those observed in face-to-face CBT including not only improvements in depressive symptoms, anxiety symptoms, and self-care behaviors but also enhancements in alleviating fear-based disorders and distress-related disorders [27, 34–36]. Research also recognized the cost-effectiveness of iCBT in treating depressive symptoms among CVD patients in comparison to an online discussion forum [35].

The nuanced findings of the present umbrella review extend beyond elucidating how differences in context and implementation across primary studies may influence the research findings. Importantly this review also identifies several critical research gaps that the attention of both researchers and clinicians. First, despite the substantial heterogeneity observed across previous RCTs, only one meta review conducted a meta-regression to explore the sources of heterogeneity and the moderators tested were limited to age, sex, and NYHA classification. Other potential clinically relevant moderators remain unexamined and should be addressed in future meta-analysis, such as CBT modalities, country-level income, heart failure etiology, comorbid conditions, and outcomes measurement approaches. Second the primary RCTs were predominately conducted in high-income countries (57.14%−90%) which calls for more clinical and research efforts in low- and middle-income countries. Third, to date no meta-analysis has quantitatively aggregated the effect sizes of internet-based CBT relative to traditional face-to-face CBT. Though we have provided a narrative synthesis of internet-based CBT in Appendix D, future meta-analytical studies are needed to quantitatively evaluate the treatment effects of digital CBT in this increasingly technology-driven healthcare context. Fourth, given the reciprocal relationship between psychological and physical outcomes in heart failure, no existing meta-analysis has quantitatively examined their potential interactions. The present umbrella review suggests that the delayed benefits in overall physical health may be attributed to the earlier improved psychological outcomes. Future RCTs should incorporate a broader range of physical outcomes (e.g., hospitalization, cardiac event-free survival, mortality rate) and future meta-analyses are encouraged to utilize advanced statistical approaches such as meta-analytic structural equation modelling to better elucidate the reciprocity.

### Limitation and Future Direction

Several limitations warrant attention. First, although the umbrella review aims to investigate therapeutic elements in CBT and compare the effectiveness between face-to-face CBT and iCBT, the included meta-analyses did not differentiate the specific therapeutic components within CBT, and evidence on the use of iCBT in HF patients is scarce. Second, nearly no meta-analyses conducted meta-regression analyses to explore potential moderators in the association between CBT and outcomes. Future empirical studies are encouraged to compare the effectiveness of different modalities of CBT in HF patients and investigate sources of heterogeneity among studies, potentially utilizing individual participant data meta-analysis. Third, the present findings may be biased towards high-income countries. More studies conducted in low- and middle-income countries (LMICs) should be carried out to enhance the geographic representativeness of the evidence in this field. Fourth, the third-wave CBT approaches that emphasize acceptance and mindfulness-based techniques were not included because the present review aimed to investigate studies involved at least one conventional form of CBT, which primarily concentrate on the modification and regulation of behaviors, cognitions, and affective states [8].

## Conclusion

The present umbrella review synthesizes the state-of-the-art knowledge on the application of CBT in HF patients. Future studies should explore how internet-based interventions can be integrated with traditional care and also investigate factors that can influence treatment adherence. More evidence is needed to address the heterogeneity between existing studies, and the application of CBT in LMICs.

## Key References

- Emmons-Bell S, Johnson C, Roth G. Prevalence, incidence and survival of heart failure: a systematic review. Heart. BMJ Publishing Group; 2022. p. 1351–60.
  - ○ This paper provides a substantive overview of heart failure as a significant public health concern.
- Zambrano J, Celano CM, Januzzi JL, Massey CN, Chung WJ, Millstein RA, et al. Psychiatric and psychological interventions for depression in patients with heart disease: A scoping review. J Am Heart Assoc. American Heart Association Inc.; 2020.
  - ○ This systematic review compares the current psychiatric and psychological interventions for treating depression in patients with heart disease. The analysis highlights the salient treatment effects of CBT in this population.
- Tolin DF. Is cognitive–behavioral therapy more effective than other therapies?: A meta-analytic review. Clin Psychol Rev. 2010;30:710–20.
  - ○ This study demonstrates CBT outperforms other psychotherapeutic interventions.
- Whiting P, Savović J, Higgins JPT, Caldwell DM, Reeves BC, Shea B, et al. ROBIS: a new tool to assess risk of bias in systematic reviews was developed. J Clin Epidemiol. 2016;69:225–34.
  - ○ This study presents the quality assessment tool used for evaluating the risk of bias in the present umbrella review.
*These are five meta-analyses included in the final synthesis.

- Chernoff RA, Messineo G, Kim S, Pizano D, Korouri S, Danovitch I, et al. Psychosocial interventions for patients with heart failure and their impact on depression, anxiety, quality of life, morbidity, and mortality: a systematic review and meta-analysis. Biopsychosocial Science and Medicine. LWW; 2022;84:560–80.
- Mhanna M, Sauer MC, Al-Abdouh A, Jabri A, Abusnina W, Safi M, et al. Cognitive behavioral therapy for depression in patients with heart failure: a systematic review and metanalysis of randomized control trials. Heart Fail Rev. Springer; 2023. p. 1091–100.
- Nso N, Emmanuel K, Nassar M, Rezaei Bookani K, Antwi-Amoabeng D, Alshamam M, et al. Efficacy of Cognitive Behavioral Therapy in Heart Failure Patients: A Systematic Review and Meta-Analysis. Cardiol Rev. Lippincott Williams and Wilkins; 2023. p. 139–48.
- Balata M, Gbreel MI, Elrashedy AA, Westenfeld R, Pfister R, Zimmer S, et al. Clinical effects of cognitive behavioral therapy in heart failure patients: a meta-analysis of randomized controlled trials. BMC Complement Med Ther. 2023;23.
- Soleimani H, Nasrollahizadeh A, Hajiqasemi M, Ebrahimzade M, Taheri H, Ebrahimi P, et al. Comparative analysis of treatment options for chronic heart failure and depression: a systematic review and Bayesian network meta-analysis. Heart Fail Rev. Springer; 2024;29:841–52.

## Supplementary Information

Below is the link to the electronic supplementary material.

Supplementary Material 1 (DOCX 60.4 KB)

## Data Availability

No datasets were generated or analysed during the current study.
