# Supplementary material for: Face-to-Face and Internet-based Cognitive Behavioral Therapy for Patients with Heart Failure: an Umbrella Review of Systematic Reviews and Meta-analyses
Source: Curr Heart Fail Rep. 2026 May 9;23(1):20. doi: 10.1007/s11897-026-00760-1 (PMC13157393; doi:10.1007/s11897-026-00760-1)
Supplement: Supplementary file 1 — Supplementary Material 1 (DOCX 60.4 KB) [file 11897_2026_760_MOESM1_ESM.docx]

**Appendix List**

[**Appendix A PRISMA 2020 Checklist** 2](#_Toc226474089)

[**Appendix B Primary studies included in the five meta-analysis** 5](#_Toc226474090)

[**Appendix C Quality assessment of included studies** 7](#_Toc226474091)

[**Appendix D iCBT study characteristics** 8](#_Toc226474092)

**Appendix A PRISMA 2020 Checklist**

| **Section and Topic** | **Item #** | **Checklist item** | **Location where item is reported** |
| --- | --- | --- | --- |
| **TITLE** | | |  |
| Title | 1 | Identify the report as a systematic review. | P1 |
| **ABSTRACT** | | |  |
| Abstract | 2 | See the PRISMA 2020 for Abstracts checklist. | P1 |
| **INTRODUCTION** | | |  |
| Rationale | 3 | Describe the rationale for the review in the context of existing knowledge. | P2 |
| Objectives | 4 | Provide an explicit statement of the objective(s) or question(s) the review addresses. | P2 |
| **METHODS** | | |  |
| Eligibility criteria | 5 | Specify the inclusion and exclusion criteria for the review and how studies were grouped for the syntheses. | P3 |
| Information sources | 6 | Specify all databases, registers, websites, organisations, reference lists and other sources searched or consulted to identify studies. Specify the date when each source was last searched or consulted. | P2 |
| Search strategy | 7 | Present the full search strategies for all databases, registers and websites, including any filters and limits used. | P2 |
| Selection process | 8 | Specify the methods used to decide whether a study met the inclusion criteria of the review, including how many reviewers screened each record and each report retrieved, whether they worked independently, and if applicable, details of automation tools used in the process. | P2 |
| Data collection process | 9 | Specify the methods used to collect data from reports, including how many reviewers collected data from each report, whether they worked independently, any processes for obtaining or confirming data from study investigators, and if applicable, details of automation tools used in the process. | P2 |
| Data items | 10a | List and define all outcomes for which data were sought. Specify whether all results that were compatible with each outcome domain in each study were sought (e.g. for all measures, time points, analyses), and if not, the methods used to decide which results to collect. | P2 |
|  | 10b | List and define all other variables for which data were sought (e.g. participant and intervention characteristics, funding sources). Describe any assumptions made about any missing or unclear information. | P2 |
| Study risk of bias assessment | 11 | Specify the methods used to assess risk of bias in the included studies, including details of the tool(s) used, how many reviewers assessed each study and whether they worked independently, and if applicable, details of automation tools used in the process. | P2 |
| Effect measures | 12 | Specify for each outcome the effect measure(s) (e.g. risk ratio, mean difference) used in the synthesis or presentation of results. | P3 |
| Synthesis methods | 13a | Describe the processes used to decide which studies were eligible for each synthesis (e.g. tabulating the study intervention characteristics and comparing against the planned groups for each synthesis (item #5)). | P2 |
|  | 13b | Describe any methods required to prepare the data for presentation or synthesis, such as handling of missing summary statistics, or data conversions. | P3 |
|  | 13c | Describe any methods used to tabulate or visually display results of individual studies and syntheses. | P3 |
|  | 13d | Describe any methods used to synthesize results and provide a rationale for the choice(s). If meta-analysis was performed, describe the model(s), method(s) to identify the presence and extent of statistical heterogeneity, and software package(s) used. | P3 |
|  | 13e | Describe any methods used to explore possible causes of heterogeneity among study results (e.g. subgroup analysis, meta-regression). | P3 |
|  | 13f | Describe any sensitivity analyses conducted to assess robustness of the synthesized results. | Appendix C |
| Reporting bias assessment | 14 | Describe any methods used to assess risk of bias due to missing results in a synthesis (arising from reporting biases). | P3 |
| Certainty assessment | 15 | Describe any methods used to assess certainty (or confidence) in the body of evidence for an outcome. | P3 |
| **RESULTS** | | |  |
| Study selection | 16a | Describe the results of the search and selection process, from the number of records identified in the search to the number of studies included in the review, ideally using a flow diagram. | P3 |
|  | 16b | Cite studies that might appear to meet the inclusion criteria, but which were excluded, and explain why they were excluded. | P3 |
| Study characteristics | 17 | Cite each included study and present its characteristics. | P3 |
| Risk of bias in studies | 18 | Present assessments of risk of bias for each included study. | P4 |
| Results of individual studies | 19 | For all outcomes, present, for each study: (a) summary statistics for each group (where appropriate) and (b) an effect estimate and its precision (e.g. confidence/credible interval), ideally using structured tables or plots. | P4 |
| Results of syntheses | 20a | For each synthesis, briefly summarise the characteristics and risk of bias among contributing studies. | P4 |
|  | 20b | Present results of all statistical syntheses conducted. If meta-analysis was done, present for each the summary estimate and its precision (e.g. confidence/credible interval) and measures of statistical heterogeneity. If comparing groups, describe the direction of the effect. | P4 |
|  | 20c | Present results of all investigations of possible causes of heterogeneity among study results. | P4 |
|  | 20d | Present results of all sensitivity analyses conducted to assess the robustness of the synthesized results. | Appendix C |
| Reporting biases | 21 | Present assessments of risk of bias due to missing results (arising from reporting biases) for each synthesis assessed. | P4 |
| Certainty of evidence | 22 | Present assessments of certainty (or confidence) in the body of evidence for each outcome assessed. | P4 |
| **DISCUSSION** | | |  |
| Discussion | 23a | Provide a general interpretation of the results in the context of other evidence. | P4 |
|  | 23b | Discuss any limitations of the evidence included in the review. | P5 |
|  | 23c | Discuss any limitations of the review processes used. | P5 |
|  | 23d | Discuss implications of the results for practice, policy, and future research. | P5 |
| **OTHER INFORMATION** | | |  |
| Registration and protocol | 24a | Provide registration information for the review, including register name and registration number, or state that the review was not registered. | P3 |
|  | 24b | Indicate where the review protocol can be accessed, or state that a protocol was not prepared. | P3 |
|  | 24c | Describe and explain any amendments to information provided at registration or in the protocol. | NA |
| Support | 25 | Describe sources of financial or non-financial support for the review, and the role of the funders or sponsors in the review. | Title page |
| Competing interests | 26 | Declare any competing interests of review authors. | Title page |
| Availability of data, code and other materials | 27 | Report which of the following are publicly available and where they can be found: template data collection forms; data extracted from included studies; data used for all analyses; analytic code; any other materials used in the review. | NA |

*From:*  Page MJ, McKenzie JE, Bossuyt PM, Boutron I, Hoffmann TC, Mulrow CD, et al. The PRISMA 2020 statement: an updated guideline for reporting systematic reviews. BMJ 2021;372:n71. doi: 10.1136/bmj.n71. This work is licensed under CC BY 4.0. To view a copy of this license, visit <https://creativecommons.org/licenses/by/4.0/>

**Appendix B Primary studies included in the five meta-analysis**

| # | Primary CBT Study | N | Chernoff 2022 | Balata 2023 | Mhanna 2023 | Nso 2023 | Soleimani 2024 |
| --- | --- | --- | --- | --- | --- | --- | --- |
| 1 | Gary et al. 2010 | 74 | Yes | — | Yes | Yes | Yes |
| 2 | Smeulders et al. 2010 | 317 | Yes | — | — | — | — |
| 3 | Dekker et al. 2012 | 41 | Yes | Yes | Yes | Yes | Yes |
| 4 | Athilingam et al. 2015 | 24 | — | — | — | Yes | — |
| 5 | Freedland et al. 2015 | 158 | Yes | Yes | Yes | Yes | Yes |
| 6 | Pressler et al. 2015 | 69 | — | — | — | Yes | — |
| 7 | Redeker et al. 2015 | 48 | Yes | — | — | — | — |
| 8 | Cajanding 2016 | 100 | — | Yes | — | — | — |
| 9 | Chang et al. 2016 | 84 | Yes | — | — | — | — |
| 10 | Lundgren et al. 2016 | 50 | Yes | — | Yes | — | — |
| 11 | Rogers et al. 2017 | 150 | — | — | — | Yes | — |
| 12 | Sherwood et al. 2017 | 139 | Yes | — | — | Yes | — |
| 13 | Peng et al. 2018 | 98 | — | — | — | Yes | — |
| 14 | Harris et al. 2019 | 39 | Yes | — | — | — | — |
| 15 | Khayati et al. 2020 | 80 | Yes | Yes | — | Yes | Yesᵃ |
| 16 | Freedland et al. 2022 | 139 | — | Yes | Yes | — | Yes^b^ |
| 17 | Hwang et al. 2022 | 46 | — | Yes | — | — | — |
| 18 | Moradi et al. 2022 | 60 | — | Yes | Yes | — | — |
|  | Total empirical studies included |  | 10 | 7 | 6 | 9 | 5 |

**Note.** ᵃ Listed as "Khayati, 2000" in Soleimani et al. but study details match Khayati 2020. ᵇ Listed as "Freedland, 2002" in Soleimani et al. but study details match Freedland 2022.

**Reference List of All Primary CBT Studies**

1. Gary RA, Dunbar SB, Higgins MK, Musselman DL, Smith AL. Combined exercise and cognitive behavioral therapy improves outcomes in patients with heart failure. J Psychosom Res. 2010;69(2):119–131.
2. Smeulders ES, van Haastregt JC, Ambergen T, Uszko-Lencer NH, Janssen-Boyne JJ, Gorgels AP, et al. Nurse-led self-management group programme for patients with congestive heart failure: randomized controlled trial. J Adv Nurs. 2010;66(7):1487–1499.
3. Dekker RL, Moser DK, Peden AR, Lennie TA. Cognitive therapy improves three-month outcomes in hospitalized patients with heart failure. J Card Fail. 2012;18(1):10–20.
4. Athilingam P, Edwards JD, Valdes EG, Ji M, Guglin M. Computerized auditory cognitive training to improve cognition and functional outcomes in patients with heart failure: results of a pilot study. Heart Lung. 2015;44(2):120–128.
5. Freedland KE, Carney RM, Rich MW, Steinmeyer BC, Rubin EH. Cognitive behavior therapy for depression and self-care in heart failure patients: a randomized clinical trial. JAMA Intern Med. 2015;175(11):1773–1782.
6. Pressler SJ, Titler M, Koelling TM, Ronfeld SM, Bull SJ, Kim SE, et al. Nurse-enhanced computerized cognitive training increases serum brain-derived neurotropic factor levels and improves working memory in heart failure. J Card Fail. 2015;21(8):630–641.
7. Redeker NS, Jeon S, Andrews L, Cline J, Jacoby D, Mohsenin V. Feasibility and efficacy of a self-management intervention for insomnia in stable heart failure. J Clin Sleep Med. 2015;11(10):1109–1119.
8. Cajanding RJM. The effectiveness of a nurse-led cognitive-behavioral therapy on the quality of life, self-esteem and mood among Filipino patients living with heart failure: a randomized controlled trial. Appl Nurs Res. 2016;31:86–93.
9. Chang YL, Chiou AF, Cheng SM, Lin KC. Tailored educational supportive care programme on sleep quality and psychological distress in patients with heart failure: a randomised controlled trial. Int J Nurs Stud. 2016;61:219–229.
10. Lundgren JG, Dahlström Ö, Andersson G, Jaarsma T, Kärner Köhler A, Johansson P. The effect of guided web-based cognitive behavioral therapy on patients with depressive symptoms and heart failure: a pilot randomized controlled trial. J Med Internet Res. 2016;18(8):e194.
11. Rogers JG, Patel CB, Mentz RJ, Granger BB, Steinhauser KE, Fiuzat M, et al. Palliative care in heart failure: the PAL-HF randomized, controlled clinical trial. J Am Coll Cardiol. 2017;70(3):331–341.
12. Sherwood A, Blumenthal JA, Koch GG, Hoffman BM, Watkins LL, Smith PJ, et al. Effects of coping skills training on quality of life, disease biomarkers, and clinical outcomes in patients with heart failure: a randomized clinical trial. Circ Heart Fail. 2017;10(1):e003410.
13. Peng X, Su Y, Hu Z, Sun X, Li X, Dolansky MA, et al. Home-based telehealth exercise training program in Chinese patients with heart failure: a randomized controlled trial. Medicine (Baltimore). 2018;97(35):e12069.
14. Harris KM, Schiele SE, Emery CF. Pilot randomized trial of brief behavioral treatment for insomnia in patients with heart failure. Heart Lung. 2019;48(5):373–380.
15. Khayati R, Rezaee N, Shakiba M, Navidian A. The effect of cognitive-behavioral training versus conventional training on self-care and depression severity in heart failure patients with depression: a randomized clinical trial. J Caring Sci. 2020;9(4):203–211.
16. Freedland KE, Skala JA, Carney RM, Steinmeyer BC, Rubin EH, Rich MW. Sequential interventions for major depression and heart failure self-care: a randomized clinical trial. Circ Heart Fail. 2022;15(5):e009422.
17. Hwang B, Granger DA, Brecht ML, Doering LV. Cognitive behavioral therapy versus general health education for family caregivers of individuals with heart failure: a pilot randomized controlled trial. BMC Geriatr. 2022;22(1):281.
18. Moradi M, Akbari M, Alavi M. The effect of cognitive-behavioral therapy on death anxiety and depression in patients with heart failure: a quasi-experimental study. Perspect Psychiatr Care. 2022;58(4):2791–2799.

**Appendix C Quality assessment of included studies**

| **Author (Year)** | **Study eligibility criteria** | | | | | **Identification and selection of studies** | | | | | **Data collection and study appraisal** | | | | | **Synthesis and findings** | | | | | | **Overall risk** |
| --- | --- | --- | --- | --- | --- | --- | --- | --- | --- | --- | --- | --- | --- | --- | --- | --- | --- | --- | --- | --- | --- | --- |
|  | Item1 | Item2 | Item3 | Item4 | Item5 | Item1 | Item2 | Item3 | Item4 | Item5 | Item1 | Item2 | Item3 | Item4 | Item5 | Item1 | Item2 | Item3 | Item4 | Item5 | Item6 |  |
| Chernoff et al. (2022) | Y | Y | Y | Y | Y | Y | Y | Y | Y | Y | Y | Y | Y | Y | Y | Y | Y | Y | Y | Y | Y | L |
| Balata et al. (2023) | Y | Y | Y | Y | PY | Y | Y | Y | Y | Y | Y | Y | Y | Y | Y | Y | Y | Y | Y | Y | Y | L |
| Mhanna et al. (2023) | Y | Y | Y | Y | Y | Y | Y | Y | Y | Y | Y | Y | Y | Y | Y | Y | Y | Y | Y | Y | Y | L |
| Nso et al. (2023) | Y | Y | Y | Y | PY | Y | Y | Y | Y | Y | Y | Y | Y | Y | NI | Y | Y | Y | PY | Y | Y | L |
| Soleimani et al. (2024) | Y | Y | Y | Y | Y | Y | Y | PY | Y | PY | Y | Y | Y | Y | Y | Y | Y | Y | N | PN | Y | L |
| ~~Peng et. al (2019)~~ | ~~Y~~ | ~~Y~~ | ~~N~~ | ~~Y~~ | ~~PY~~ | ~~Y~~ | ~~Y~~ | ~~N~~ | ~~NI~~ | ~~Y~~ | ~~Y~~ | ~~Y~~ | ~~Y~~ | ~~Y~~ | ~~NI~~ | ~~Y~~ | ~~Y~~ | ~~Y~~ | ~~PY~~ | ~~Y~~ | ~~Y~~ | ~~H~~ |

Note. yes (Y); probably yes (PY); no information (NI); probably no (PN); no (N); L (low risk); M (moderate risk); H (high risk)

**Appendix D iCBT study characteristics**

To address the knowledge deficit about subgroup analyses to assess the efficacy of internet-based cognitive behavioral therapy (iCBT), we extracted all five empirical studies on iCBT from the reference list for further examination. Four of these studies originated from the same research project in Sweden, which evaluated an internet-based CBT intervention incorporating psychoeducation and homework assignments. The fifth study was a research protocol for a commercially available digital CBT program called Daylight in the United States. We extracted the following information. ***Study characteristics*** included country, female percentage, age range, study design, HF condition (e.g., HF frequency, preserved, mid-range, or reduced), medication history, comorbid condition. ***Intervention group characteristics*** included sample size, intervention delivery modality, guidance involvement (e.g., no guidance, synchronous/real-time guidance, or asynchronous/delayed guidance), intervention duration (session number). ***Control group characteristics*** included samples size and control type. ***Outcome characteristics*** included types of psychiatric outcome, types of behavioral outcome, and type of cardiac or medical outcome; ***Main findings*** included overall effectiveness of the interventions in improving relevant outcomes for patients with HF and its corresponding timepoints (i.e., post-intervention). ***Therapeutic elements of CBT*** included behavioral modification, cognitive restructuring, problem solving, psychoeducation, and mindfulness.

The Swedish study was a two-arm RCT comparing a 9-session, 7-module iCBT program to an active control of an online discussion forum on HF self-care. Participants had a mean age of 63 years (SD = 12), with 41% being female. Attrition rate on average was 18% and the follow-up duration ranged from 3 weeks to 12 months. Morbidity included hypertension, diabetes, pulmonary disease, Stroke/TIA, renal disease, and cancer, and all received medication for HF. iCBT were synchronous/real-time guided. Therapeutic elements of iCBT included behavioral modification, problem solving, and psychoeducation. Outcomes measured across the four Swedish studies encompassed depressive symptoms, self-care behaviors, distress, fear disorder, and the cost-effectiveness of iCBT. Results from the four RCTs did not reveal any statistically significant differences in self-care outcomes between the patients who received iCBT and those in the online discussion group control condition, at both the 3-week and 9-week follow-up. However, the improvement in depressive symptoms was found to be significantly associated with improvements in autonomy-related self-care behaviors. Additionally, the iCBT intervention was successful in reducing symptoms of distress-related disorders, and it also demonstrated a lesser degree of efficacy in alleviating symptoms of fear-based disorders [32,34–36].

In contrast, the Daylight research protocol [31] in the United States employed an asynchronous (delayed) iCBT approach guided by an animated therapist, consisting of four sessions with a waitlist control design. Therapeutic elements in this protocol included behavioral modification, cognitive restructuring, and mindfulness. The proposed outcomes focused on anxiety symptoms, depressive symptoms, sleep difficulties, quality of life, anxiety sensitivity, worry severity, and shame. However, no effectiveness data were reported in this study as it remained a protocol.
